# Supplementary material for: Minigene Splicing Assays Identify 20 Spliceogenic Variants of the Breast/Ovarian Cancer Susceptibility Gene RAD51C
Source: Cancers (Basel). 2022 Jun 15;14(12):2960. doi: 10.3390/cancers14122960 (PMC9221245; doi:10.3390/cancers14122960)
Supplement: Supplementary file 1 [file cancers-14-02960-s001.zip › Supplementary_Figure_S1 - Protocol Pipeline.pdf]

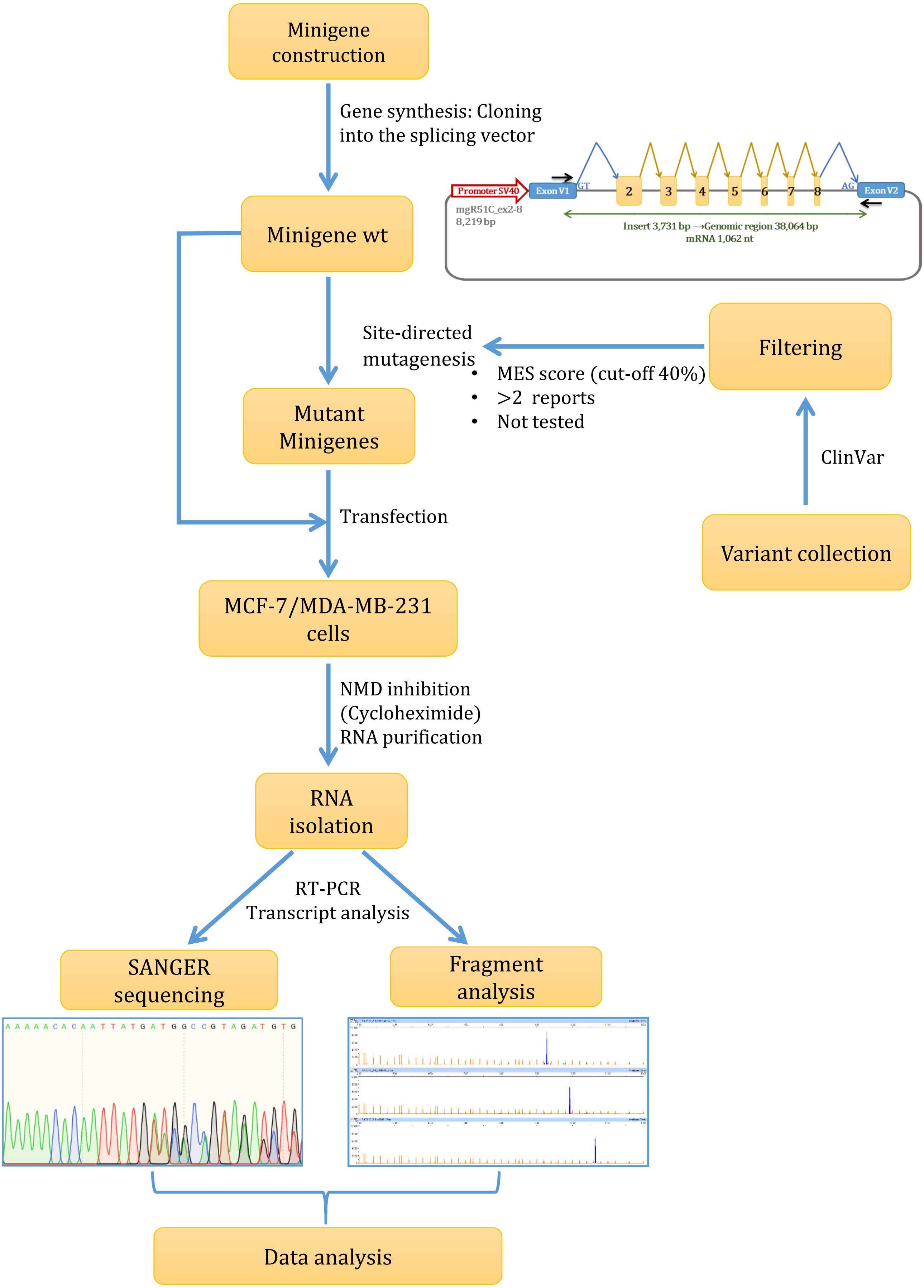

Supplementary Figure S1. Workflow of the minigene protocol. Basic protocol for the analysis of canonical BRIDGES variants. The basic assay includes the following steps: (1) Minigene construction; (2) Site-directed mutagenesis; (3) Transfection of the wild type and mutant minigenes and inhibition of Nonsense-mediated decay and RNA purification; (4) Transcript sequencing and fragment analysis by fluorescent capillary electrophoresis; (5) Data analysis.
