# Supplementary material for: Minigene Splicing Assays Identify 20 Spliceogenic Variants of the Breast/Ovarian Cancer Susceptibility Gene RAD51C
Source: Cancers (Basel). 2022 Jun 15;14(12):2960. doi: 10.3390/cancers14122960 (PMC9221245; doi:10.3390/cancers14122960)
Supplement: Supplementary file 1 [file cancers-14-02960-s001.zip › Supplementary_Figure_S2 - RAD51C protein alignment.pdf]

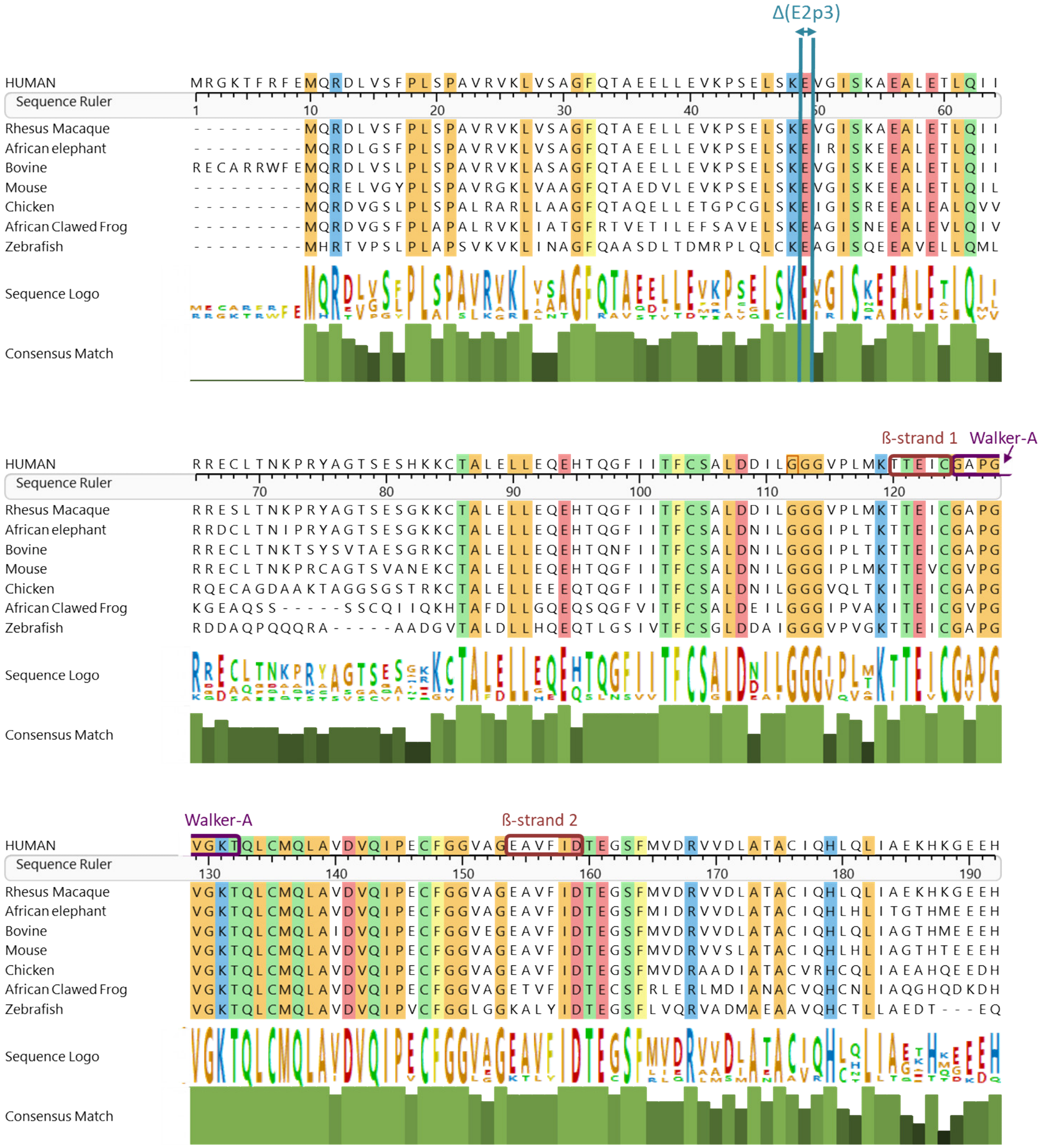

Supplementary Figure S2. Alignment and amino acid conservation of the RAD51C protein (I). Amino acid conservation of the deleted in-frame sequences of the anomalous *RAD51C* transcripts Δ(E2p3) and Δ(E5). The deleted in-frame regions are marked in blue stripes and arrows and the key functional domains of RAD51C in boxes of different colors (the Walker domains for ATP-hydrolysis are in purple, the β-strands are in brown and the nuclear localization signal is in gray). Protein sequences were aligned with the Align tool of the Uniprot database (https://www.uniprot.org/align/). The alignment file was visualized with MegAlign Pro version 17.1.1 of DNASTAR's Lasergene software. The conserved residues are highlighted (nonpolar amino acids in orange, polar amino acids in green, polar basic amino acids in blue and polar acidic amino acids in red) and the color intensity of green bars above the protein sequence indicates the degree of conservation of each amino acid. Organisms: Human (*Homo sapiens*); Rhesus Macaque (*Macaca mulatta*); African elephant (*Loxodonta africana*); Bovine (*Bos taurus*); Mouse (*Mus musculus*); Chicken (*Gallus gallus*); African Clawed Frog (*Xenopus tropicalis*); Zebrafish (*Danio rerio*).

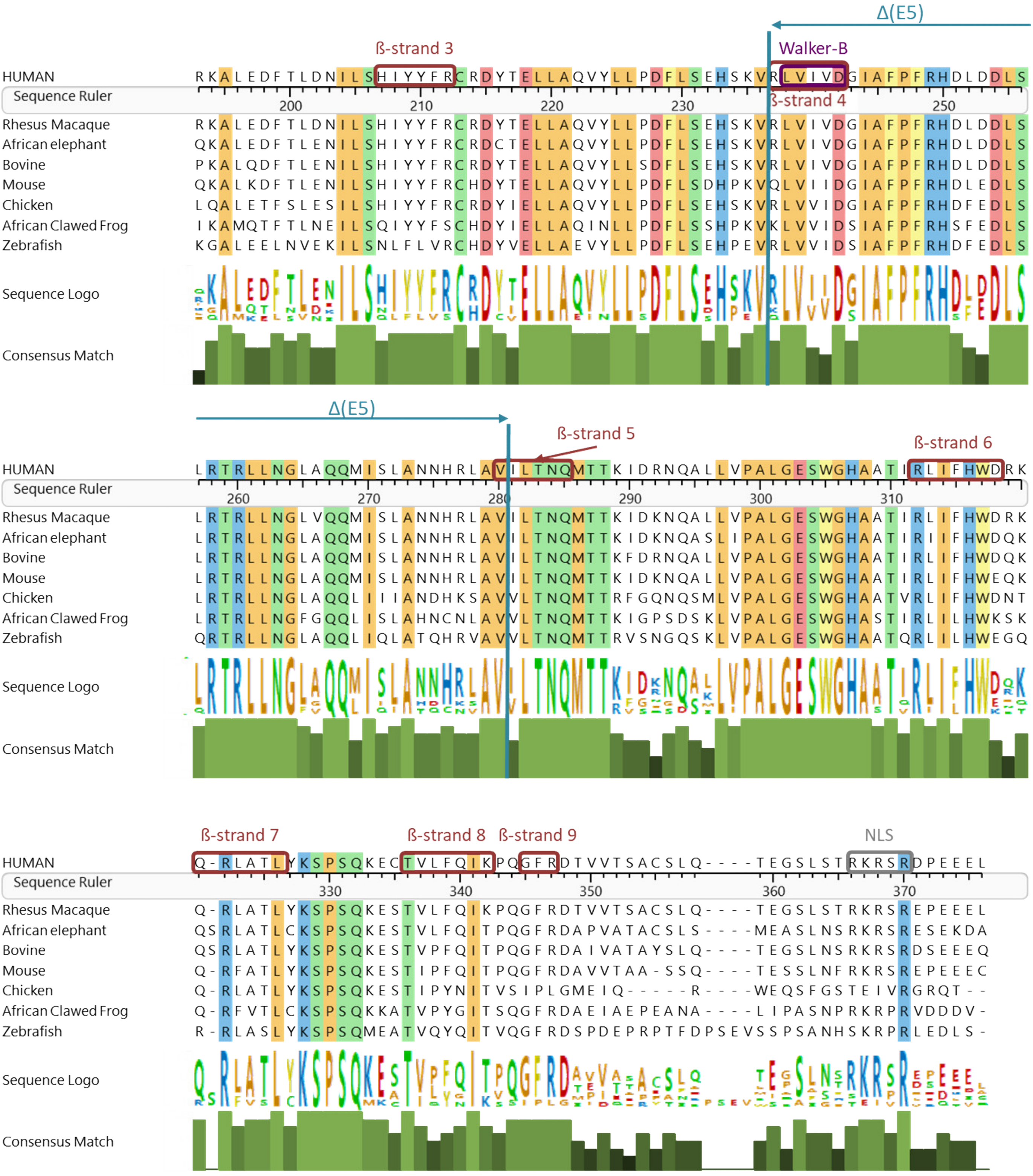

Supplementary Figure S2. Alignment and amino acid conservation of the RAD51C protein (II).
