# Supplementary material for: Minigene Splicing Assays Identify 20 Spliceogenic Variants of the Breast/Ovarian Cancer Susceptibility Gene RAD51C
Source: Cancers (Basel). 2022 Jun 15;14(12):2960. doi: 10.3390/cancers14122960 (PMC9221245; doi:10.3390/cancers14122960)
Supplement: Supplementary file 1 [file cancers-14-02960-s001.zip › Supplementary_Table_S1-Bioinformatics analysis ClinVar.pdf]

Supplementary Table S1. Bioinformatics analysis of *RAD51C* variants with Max Ent Score.

RAD51C VARIANTS[1]	EXON/ INTRON	MAX ENT SCORE ANALYSIS							SpliceAI ANALYSIS[6]
		MES wt	MES mut	MES score change[2]	MES cryptic SS[3]	MES *de novo* SS-wt[4]	MES *de novo* SS-mut[4]	MES score change[2,5]	
c.146-8A>C	ivs1/Ex2	9.5	10.8	+14.4%	28-nt upstream 6.5				
c.146-8A>G	ivs1/Ex2	9.5	9.4	-0.2%	28-nt upstream 6.5				
c.146-7C>G (1 Report)	ivs1/Ex2	9.5	3.1	-67.0%	28-nt upstream 6.5	1.8	9.9	+436.6% 6-nt upstream	AL: 0.90 AG: 0.95→6-nt upstream
c.146-4T>C	ivs1/Ex2	9.5	9.1	-3.3%	28-nt upstream 6.5				
c.146-4_146-2del (2 Reports)	ivs1/Ex2	9.5	-0.8	-108.6%	28-nt upstream 6.5	0.3	5.9	+2217.2% 3-nt downstream	AL: 0.93
c.146-3C>G (2 Reports)	ivs1/Ex2	9.5	1.9	-80.4%	28-nt upstream 6.5				AL: 0.81 DL: 0.38→259-nt downstream
c.146-3C>T	ivs1/Ex2	9.5	8.7	-8.4%	28-nt upstream 6.5				
c.146-2A>G (1 Report)	ivs1/Ex2	9.5	1.5	-84.1%	28-nt upstream 6.5	-5.5	3.3	+160.2% 1-nt upstream(CG)	AL: 0.93
c.146-1G>T (1 Report)	ivs1/Ex2	9.5	0.9	-90.9%	28-nt upstream 6.5				AL: 0.93 DL:0.42→259-nt downstream
c.146-1G>A (1 Report)	ivs1/Ex2	9.5	0.7	-92.5%	28-nt upstream 6.5	0.3	4.0	+1464.6% 3-nt downstream	AL: 0.93 DL:0.28→259-nt downstream
c.402A>G	Ex2	4.8	3.0	-37.8%	27-nt downstream 5.4				
c.403T>C	Ex2	4.8	6.5	+36.3%	27-nt downstream 5.4				
c.404G>T (1 Report) (Neidhardt et al., 2017)	Ex2	4.8	-4.5	-193.0%	27-nt downstream 5.4				AL:0.35→256-nt upstream DL: 0.92 DG: 0.37→22-nt upstream
c.404G>C (5 Reports)	Ex2	4.8	-6.6	-237.2%	27-nt downstream 5.4				AL:0.31→256-nt upstream DL: 0.94

RAD51C VARIANTS[1]	EXON/ INTRON	MAX ENT SCORE ANALYSIS							SpliceAI ANALYSIS[6]
		MES wt	MES mut	MES score change[2]	MES cryptic SS[3]	MES *de novo* SS-wt[4]	MES *de novo* SS-mut[4]	MES score change[2,5]	
(Neidhardt et al., 2017)									DG: 0.29→22-nt upstream
c.404G>A (6 Reports) (Sanoguera-Miralles et al., 2020)	Ex2	4.8	-3.5	-172.8%	27-nt downstream 5.4				AL:0.40→256-nt upstream DL: 0.85 DG: 0.25→22-nt upstream
c.404+1G>C (1 Report)	Ex2/ivs2	4.8	-3.5	-172.6%	27-nt downstream 5.4				AL:0.33→256-nt upstream DL: 0.97 DG: 0.30→22-nt upstream
c.404+1G>A (1 Report)	Ex2/ivs2	4.8	-3.4	-170.7%	27-nt downstream 5.4				AL:0.35→256-nt upstream DL: 0.97 DG: 0.27→22-nt upstream
c.404+6_404+10del	Ex2/ivs2	4.8	5.4	+12.5%	27-nt downstream 5.4				
c.404+2T>C (5 Reports)	Ex2/ivs2	4.8	-3.0	-161.8%	27-nt downstream 5.4				AL:0.29→256-nt upstream DL: 0.97 DG: 0.52→27-nt downstream
c.404+3A>G (3 Reports)	Ex2/ivs2	4.8	0.6	-88.3%	27-nt downstream 5.4				AL:0.27→256-nt upstream DL: 0.61 DG: 0.48→27-nt downstream
c.404+7_404+10del	Ex2/ivs2	4.8	4.8	0%	27-nt downstream 5.4				DG: 0.21→27-nt downstream
c.404+6A>G	Ex2/ivs2	4.8	5.4	+12.5%	27-nt downstream 5.4				
c.404+7T>C	Ex2/ivs2	4.8	4.8	0%	27-nt downstream 5.4				
c.405-10C>G	Ex2/ivs2	7.7	6.6	-14.4%					
c.405-6dup	ivs2/Ex3	7.7	7.3	-5.1%					
c.405-8G>A	ivs2/Ex3	7.7	7.6	-1.0%					
c.405-6T>A (2 Reports) (Sanoguera-Miralles et al., 2020)	ivs2/Ex3	7.7	2.2	-72.1%		0.2	8.6	+4549.3% 4-nt upstream	AL: 0.64 AG: 0.95→4-nt upstream
c.405-5G>A	ivs2/Ex3	7.7	8.7	+13.1%					
c.405-3C>T	ivs2/Ex3	7.7	6.6	-14.2%					

RAD51C VARIANTS[1]	EXON/ INTRON	MAX ENT SCORE ANALYSIS							SpliceAI ANALYSIS[6]
		MES wt	MES mut	MES score change[2]	MES cryptic SS[3]	MES *de novo* SS-wt[4]	MES *de novo* SS-mut[4]	MES score change[2,5]	
c.405-2A>G (1 Report)	ivs2/Ex3	7.7	-0.2	-103.1%					AL: 0.99 DL:0.41→167-nt downstream AG: 0.64→7-nt downstream
c.405-1G>A (1 Report)	ivs2/Ex3	7.7	-1.0	-113.4%		-2.5	3.3	+233.0% 7-nt downstream	AL:0.99 DL:0.52→167-nt downstream AG:0.52→7-nt downstream
c.405-1G>C (3 Reports)	ivs2/Ex3	7.7	-0.4	-104.5%		-2.5	4.2	+272.6% 7-nt downstream	AL:0.99 DL:0.41→167-nt downstream AG:0.67→7-nt downstream
c.405T>C	Ex3	7.7	8.0	+3.9%					
c.406A>T	Ex3	7.7	9.2	+19.0%					
c.570G>A	Ex3	10.5	11.0	+5.2%	42-nt downstream 3.6				
c.571+1del (4 Reports)	Ex3/ivs3	10.5	-14.1	-234.9%	42-nt downstream 3.6	-6.5	11.1	+269.7% 1-nt upstream	DL:0.99 DG:0.99→1-nt upstream
c.571G>A	Ex3	10.5	9.4	-10.5%	42-nt downstream 3.6				AL: 0.26→167-nt upstream DL:0.21
c.571+1G>T (1 Report)	Ex3/ivs3	10.5	2.0	-81.4%	42-nt downstream 3.6				AL: 0.96→167-nt upstream DL:0.99
c.571+2T>A (1 Report)	Ex3/ivs3	10.5	2.3	-78.3%	42-nt downstream 3.6	0.9	4.2	+382.9% 4-nt downstream	AL: 0.97→167-nt upstream DL:0.99
c.571+4A>G	Ex3/ivs3	10.5	8.1	-22.5%	42-nt downstream 3.6	0.9	5.5	+526.2% 4-nt downstream	AL: 0.47→167-nt upstream DL:0.38 DG:0.24→4-nt downstream
c.571+5G>A	Ex3/ivs3	10.5	5.8	-44.2%	42-nt downstream 3.6				AL: 0.93→167-nt upstream

RAD51C VARIANTS[1]	EXON/ INTRON	MAX ENT SCORE ANALYSIS							SpliceAI ANALYSIS[6]
		MES wt	MES mut	MES score change[2]	MES cryptic SS[3]	MES *de novo* SS-wt[4]	MES *de novo* SS-mut[4]	MES score change[2,5]	
(4 Reports) (Sanoguera-Miralles et al., 2020)									DL:0.90
c.571+6T>G	Ex3/ivs3	10.5	9.2	-12.4%	42-nt downstream 3.6	-2.8	5.7	+302.6% 5-nt downstream	AL: 0.25→167-nt upstream DL:0.25 DG:0.42→5-nt downstream
c.571+7T>C	Ex3/ivs3	10.5	10.5	0%	42-nt downstream 3.6				
c.571+9G>C	Ex3/ivs3	10.5	10.5	0%	42-nt downstream 3.6				
c.572-7G>A	ivs3/Ex4	7.4	5.9	-21.3%	33-nt upstream 6.4				
c.572-3C>T	ivs3/Ex4	7.4	7.4	-0.7%	33-nt upstream 6.4				
c.572-3C>G (3 Reports)	ivs3/Ex4	7.4	-1.4	-118.3%	33-nt upstream 6.4				AL: 0.99 DL: 0.71→134-nt downstream
c.572-2A>G (2 Reports)	ivs3/Ex4	7.4	-0.5	-107.1%	33-nt upstream 6.4				AL: 0.99 DL: 0.65→134-nt downstream
c.572-1G>T (1 Report) (Sanoguera-Miralles et al., 2020)	ivs3/Ex4	7.4	-1.2	-115.8%	33-nt upstream 6.4				AL: 0.99 DL: 0.71→134-nt downstream
c.572-1G>C (5 Reports)	ivs3/Ex4	7.4	-0.6	-108.6%	33-nt upstream 6.4				AL: 0.99 DL: 0.73→134-nt downstream
c.704dup	Ex4	9.1	9.1	0%	22-nt downstream 5.9				
c.704del	Ex4	9.1	9.1	0%	22-nt downstream 5.9				
c.703A>G	Ex4	9.1	8.6	-6.1%	22-nt downstream 5.9				
c.704A>C	Ex4	9.1	8.5	-6.2%	22-nt downstream 5.9				
c.705G>T	Ex4	9.1	2.6	-72.0%	22-nt downstream 5.9				AL: 0.69→134-nt upstream

RAD51C VARIANTS[1]	EXON/ INTRON	MAX ENT SCORE ANALYSIS							SpliceAI ANALYSIS[6]
		MES wt	MES mut	MES score change[2]	MES cryptic SS[3]	MES *de novo* SS-wt[4]	MES *de novo* SS-mut[4]	MES score change[2,5]	
(8 Reports) (Sanoguera-Miralles et al., 2020)									DL: 0.87
c.705+1G>T (1 Report)	Ex4/ivs4	9.1	0.6	-93.4%	22-nt downstream 5.9				AL: 0.80→134-nt upstream DL: 1.00
c.705+1G>A (3 Reports)	Ex4/ivs4	9.1	0.9	-89.9%	22-nt downstream 5.9				AL: 0.73→134-nt upstream DL: 1.0
c.705+3A>T (2 Reports)	Ex4/ivs4	9.1	5.1	-43.6%	22-nt downstream 5.9				AL: 0.25→134-nt upstream DL: 0.32
c.705+3A>G (5 Reports)	Ex4/ivs4	9.1	4.6	-49.6%	22-nt downstream 5.9	-2.1	6.1	+383.6% 2-nt downstream	DG:0.44→2-nt downstream
c.705+4T>C	Ex4/ivs4	9.1	9.8	+7.4%	22-nt downstream 5.9				
c.705+4T>G	Ex4/ivs4	9.1	9.5	+3.8%	22-nt downstream 5.9				
c.705+8T>A	Ex4/ivs4	9.1	9.1	0%	22-nt downstream 5.9				
c.705+9C>G	Ex4/ivs4	9.1	9.1	0%	22-nt downstream 5.9				
c.705+9C>T	Ex4/ivs4	9.1	9.1	0%	22-nt downstream 5.9				
c.705+10A>G	Ex4/ivs4	9.1	9.1	0%	22-nt downstream 5.9				
c.706-9T>A (1 Report)	ivs4/Ex5	11.1	4.0	-64.2%	52-nt downstream 5.5	-2.9	5.5	+355.8% 7-nt upstream	AL:0.67 AG:0.96→7-nt upstream
c.706-9T>C	ivs4/Ex5	11.1	11.8	+6.6%	52-nt downstream 5.5				
c.706-7T>C	ivs4/Ex5	11.1	10.4	-6.4%	52-nt downstream 5.5				
c.706-6A>G	ivs4/Ex5	11.1	11.2	+1.1%	52-nt downstream 5.5				
c.706-4T>A	ivs4/Ex5	11.1	9.6	-13.2%	52-nt downstream 5.5				

RAD51C VARIANTS[1]	EXON/ INTRON	MAX ENT SCORE ANALYSIS							SpliceAI ANALYSIS[6]
		MES wt	MES mut	MES score change[2]	MES cryptic SS[3]	MES *de novo* SS-wt[4]	MES *de novo* SS-mut[4]	MES score change[2,5]	
c.706-2A>C (3 Reports) (Sanoguera-Miralles et al., 2020)	ivs4/Ex5	11.1	3.1	-72.5%	52-nt downstream 5.5	-2.1	3.3	+262.3% 10-nt downstream	AL:1.00 DL: 0.27→132-nt downstream AG:0.70→10-nt downstream
c.706-2A>G (17 Reports) (Sanoguera-Miralles et al., 2020)	ivs4/Ex5	11.1	3.1	-71.7%	52-nt downstream 5.5	-2.1	3.2	+254.5% 10-nt downstream	AL:1.00 DL: 0.42→132-nt downstream AG:0.54→10-nt downstream
c.706-1G>A (1 Report)	ivs4/Ex5	11.1	2.4	-78.9%	52-nt downstream 5.5	-0.8	7.2	+1028.9% 1-nt downstream	AL:1.00 AG:0.74→1-nt downstream
c.706-1G>T (2 Reports)	ivs4/Ex5	11.1	2.5	-77.5%	52-nt downstream 5.5	-2.1	4.3	+308.0% 10-nt downstream	AL:1.00 AG:0.77→10-nt downstream
c.707T>A	Ex5	11.1	11.0	-0.9%	52-nt downstream 5.5				
c.835G>C	Ex5	8.6	7.5	-12.1%					
c.836C>G	Ex5	8.6	8.1	-5.8%					
c.837+1G>C (2 Reports)	Ex5/ivs5	8.6	0.3	-96.6%					AL:0.77→132-nt upstream DL:1.00
c.837+2dup (1 Report)	Ex5/ivs5	8.6	-20.4	-337.6%					AL:0.44→132-nt upstream DL:0.91
c.837+1del (2 Reports)	Ex5/ivs5	8.6	-29.1	-440.1%					AL:0.57→132-nt upstream DL:1.00
c.837+1G>T (4 Reports)	Ex5/ivs5	8.6	0.1	-99.3%					AL:0.77→132-nt upstream DL:1.00
c.837+1G>A (5 Reports) (Pelttari et al., 2011)	Ex5/ivs5	8.6	0.4	-95.5%					AL:0.77→132-nt upstream DL:1.00
c.837+4_837+7del (3 Reports)	Ex5/ivs5	8.6	-8.9	-203.9%					AL:0.70→132-nt upstream DL:1.00
c.837+2T>C (3 Reports) (Sanoguera-Miralles et al., 2020)	Ex5/ivs5	8.6	0.8	-90.5%					AL:0.67→132-nt upstream DL:0.99
c.837+4del	Ex5/ivs5	8.6	-12.4	-245.3%					AL:0.67→132-nt upstream

RAD51C VARIANTS[1]	EXON/ INTRON	MAX ENT SCORE ANALYSIS							SpliceAI ANALYSIS[6]
		MES wt	MES mut	MES score change[2]	MES cryptic SS[3]	MES *de novo* SS-wt[4]	MES *de novo* SS-mut[4]	MES score change[2,5]	
(1 Report)									DL:1.00
c.837+5G>T (1 Report)	Ex5/ivs5	8.6	-0.9	-110.7%					AL:0.75→132-nt upstream DL:0.99
c.837+8_837+10dup	Ex5/ivs5	8.6	8.6	0%					
c.837+5G>C (1 Report)	Ex5/ivs5	8.6	3.3	-62.1%					AL:0.75→132-nt upstream DL:1.00
c.837+7A>G	Ex5/ivs5	8.6	8.6	0%					
c.838-9T>G	ivs5/Ex6	10.2	9.3	-8.5%					
c.838-6T>C	ivs5/Ex6	10.2	10.6	+4.3%					
c.838-5T>C	ivs5/Ex6	10.2	9.4	-8.0%					
c.838-2A>T (1 Report)	ivs5/Ex6	10.2	1.8	-82.3%		2.0	3.4	+74.4% 18-nt downstream	AL:0.97 DL:0.93→67-nt downstream
c.838-2A>G (2 Reports)	ivs5/Ex6	10.2	2.2	-78.3%					AL:0.97 DL:0.93→67-nt downstream
c.838G>A	Ex6	10.2	9.3	-8.1%					
c.904G>C (1 Report)	Ex6	5.6	1.8	-68.1%	4-nt downstream 6.2				AL: 0.33→67-nt upstream DL:0.57 DG:0.57→4-nt downstream
c.904G>A (4 Reports)	Ex6	5.6	1.0	-82.1%	4-nt downstream 6.2				AL: 0.49→67-nt upstream DL:0.62 DG:0.36→4-nt downstream
c.904+1G>A (1 Report)	Ex6/ivs6	5.6	-2.6	-147.2%	4-nt downstream 6.2				AL: 0.46→67-nt upstream DL:0.98 DG:0.38→4-nt downstream
c.904+1G>T (3 Reports)	Ex6/ivs6	5.6	-3.0	-153.0%	4-nt downstream 6.2				AL: 0.65→67-nt upstream DL:0.98
c.904+3G>T	Ex6/ivs6	5.6	4.4	-21.0%	4-nt downstream 5.7				
c.904+4G>T	Ex6/ivs6	5.6	4.3	-22.6%					AL: 0.20→67-nt upstream
c.904+5G>T	Ex6/ivs6	5.6	-0.9	-116.8%					AL:0.95→67-nt upstream

RAD51C VARIANTS[1]	EXON/ INTRON	MAX ENT SCORE ANALYSIS							SpliceAI ANALYSIS[6]
		MES wt	MES mut	MES score change[2]	MES cryptic SS[3]	MES *de novo* SS-wt[4]	MES *de novo* SS-mut[4]	MES score change[2,5]	
(13 Reports) (Meindl et al., 2010)									DL:0.94
c.904+6T>C	Ex6/ivs6	5.6	5.3	-5.3%					AL:0.55→67-nt upstream DL:0.46
c.904+7A>C	Ex6/ivs6	5.6	5.6	0%					
c.905-7C>T	ivs6/Ex7	8.2	8.3	+1.0%					
c.905-5C>T	ivs6/Ex7	8.2	9.8	+20.0%					
c.905-5C>G	ivs6/Ex7	8.2	6.6	-19.4%					AL:0.25 DL:0.39→61-nt downstream
c.905-4T>G	ivs6/Ex7	8.2	8.8	+7.5%					
c.905-3_906del (3 Reports)	ivs6/Ex7	8.2	-8.6	-204.7%		-5.9	4.5	+176.9% 7-nt downstream	AL:0.91 DL:0.88→61-nt downstream
c.905-2A>G (4 Reports) (Coulet et al., 2013)	ivs6/Ex7	8.2	0.2	-97.2%					AL:0.91 DL:0.89→61-nt downstream
c.905-2del (4 Reports)	ivs6/Ex7	8.2	2.1	-74.6%					AL:0.91 DL:0.87→61-nt downstream
c.905-2A>C (4 Reports) (Sanoguera-Miralles et al., 2020)	ivs6/Ex7	8.2	0.1	-98.3%					AL:0.91 DL:0.85→61-nt downstream
c.905-2_905-1del (10 Reports) (Sanoguera-Miralles et al., 2020)	ivs6/Ex7	8.2	-7.9	-195.9%					AL:0.91 DL:0.88→61-nt downstream
c.905-1G>A (1 Report)	ivs6/Ex7	8.2	-0.6	-106.9%		-1.3	6.7	+634.4% 1-nt downstream	AL:0.91 DL:0.25→61-nt downstream AG:0.59→1-nt downstream
c.905G>T	Ex7	8.2	6.9	-15.5%					AL:0.32 DL:0.42→61-nt downstream

RAD51C VARIANTS[1]	EXON/ INTRON	MAX ENT SCORE ANALYSIS							SpliceAI ANALYSIS[6]
		MES wt	MES mut	MES score change[2]	MES cryptic SS[3]	MES *de novo* SS-wt[4]	MES *de novo* SS-mut[4]	MES score change[2,5]	
c.906G>A	Ex7	8.2	7.6	-6.9%					AG:0.24→3-nt downstream
c.964del	Ex7	8.7	8.7	0%					
c.964A>C	Ex7	8.7	8.0	-7.6%					
c.965G>A (1 Report)	Ex7	8.7	2.0	-77.2%					AL:0.82→61-nt upstream DL:0.91
c.965G>C	Ex7	8.7	5.6	-36.1%					AL:0.77→61-nt upstream DL:0.88
c.965+1G>A (3 Reports)	Ex7/ivs7	8.7	0.5	-94.3%					AL:0.86→61-nt upstream DL:0.94
c.965+4A>G	Ex7/ivs7	8.7	7.1	-17.8%		-6.6	3.7	+156.0% 4-nt downstream	DL:0.21
c.965+5G>A (3 Reports) (Sanoguera-Miralles et al., 2020)	Ex7/ivs7	8.7	3.8	-56.5%					AL:0.83→61-nt upstream DL:0.92
c.965+7A>G	Ex7/ivs7	8.7	8.7	0%					
c.965+8C>G	Ex7/ivs7	8.7	8.7	0%					
c.965+10G>A	Ex7/ivs7	8.7	8.7	0%					
c.966-8T>G	ivs7/Ex8	7.3	6.7	-7.9%					AL:0.46→3-nt upstream DL:0.29→61-nt downstream
c.966-4G>A	ivs7/Ex8	7.3	10.7	+46.8%					AL:0.74→3-nt upstream
c.966-4G>C	ivs7/Ex8	7.3	9.3	+28.4%					AL: 0.74→3-nt upstream
c.966-3C>T	ivs7/Ex8	7.3	6.5	-10.5%	3-nt upstream 4.6				AL: 0.45 DL:0.41→61-nt downstream
c.966-2A>G (1 Report) (Sanoguera-Miralles et al., 2020)	ivs7/Ex8	7.3	-0.7	-109.7%	3-nt upstream 7.0				AL: 0.84 DL:0.66→61-nt downstream
c.966del	Ex8	7.3	5.5	-24.8%	3-nt upstream 5.7				AL:0.84 DL:0.48→61-nt downstream

RAD51C VARIANTS[1]	EXON/ INTRON	MAX ENT SCORE ANALYSIS							SpliceAI ANALYSIS[6]
		MES wt	MES mut	MES score change[2]	MES cryptic SS[3]	MES *de novo* SS-wt[4]	MES *de novo* SS-mut[4]	MES score change[2,5]	
									AG:0.40→1-nt downstream
**c.966-1G>C** (2 Reports)	ivs7/Ex8	7.3	-0.8	-111.2%	3-nt upstream 6.8				AL: 0.84 DL:0.56→61-nt downstream
**c.966-1G>T** (2 Reports)	ivs7/Ex8	7.3	-1.4	-118.6%	3-nt upstream 7.2				AL: 0.84 DL:0.62→61-nt downstream
**c.966-1G>A** (2 Reports)	ivs7/Ex8	7.3	-1.5	-120.6%	3-nt upstream 6.5				AL: 0.84 DL:0.65→61-nt downstream
c.966G>A	Ex8	7.3	6.7	-7.4%	3-nt upstream 5.7				
c.966G>T	Ex8	7.3	5.2	-27.7%	3-nt upstream 5.7				AL:0.49 DL:0.44→61-nt downstream
c.967T>C	Ex8	7.3	6.5	-10.8%	3-nt upstream 5.7				
**c.1026+1G>C** (1 Report)	Ex8/ivs8	2.0	-6.3	-417.1%	44-nt downsteam 6.8				AL:0.72→61-nt upstream DL:0.88
**c.1026+5_1026+7del** (12 Reports) (Sanoguera-Miralles et al., 2020)	Ex8/ivs8	2.0	-6.3	-417.3%	44-nt downsteam 6.8				AL:0.74→61-nt upstream DL:0.88
c.1026+9T>G	Ex8/ivs8	2.0	2.0	0%	44-nt downsteam 6.8				

[1]The variants selected after bioinformatics analysis are in blue and bold (MES score ≥40%). For selected variants, the number of reports in the ClinVar database is indicated in brackets and, if applicable, the citation of the article in which it has been functionally tested is also provided. The green cells contain the variants finally selected after applying all filters (MES score ≥40%, one variant per splice site position, more than two reports in the ClinVar database and not previously functionally tested). [2]MES score changes (Δ%), wildtype(wt) vs. mutant(mut). [3]MES score of cryptic splice sites (60-nt upstream and 60-nt downstream of the canonical splice site were analyzed) (MES≥3). [4]*De novo*: predicted creation of new alternative splice sites (MES≥3). [5]When the use of a non-canonical splice site (other than the classical GT-AG) is predicted, it is indicated in parentheses. [6]SpliceAI parameters were as follows : a)genome version → hg38; b)score type → raw; c)max distance → 10000 nt; d)Illumina's pre-computed scores → yes. Scores (≥0.2) and positions of acceptor loss (AL), donor loss (DL), acceptor gain (AG) and donor gain (DG) are shown.
