# Supplementary material for: Minigene Splicing Assays Identify 20 Spliceogenic Variants of the Breast/Ovarian Cancer Susceptibility Gene RAD51C
Source: Cancers (Basel). 2022 Jun 15;14(12):2960. doi: 10.3390/cancers14122960 (PMC9221245; doi:10.3390/cancers14122960)
Supplement: Supplementary file 1 [file cancers-14-02960-s001.zip › Supplementary_Table_S2-RAD51C Mutagenesis primers ClinVar.pdf]

Supplementary Table S2. Mutagenesis primers for *RAD51C* variants.

Variant	Exon/Intron	Primers (5'→3')
c.146-4_146-2del	ivs1	GGGTTCTTTTTTTCTTATTTTACTTGAAGTTGGGATATCTAAAGCAGAAG
		CTTCTGCTTTAGATATCCCAACTTCAAGTAAAATAAGAAAAAAAGAACCC
c.146-3C>G	ivs1	TTTTTTCTTATTTTACTTTGAGAAGTTGGGATATCTAAA
		TTTAGATATCCCAACTTCTCAAAGTAAAATAAGAAAAAA
c.404+2T>C	ivs2	TGGAAAAACACAATTATGGCAAAATAAAGTGTTCTCCTT
		AAGGAGAACACTTTATTTTGCCATAATTGTGTTTTTCCA
c.404+3A>G	ivs2	GGAAAAACACAATTATGGTGAAATAAAGTGTTCTCCTTT
		AAAGGAGAACACTTTATTTCACCATAATTGTGTTTTTCC
c.405-1G>C	ivs2	TGTCATCTTTCTGTTGACACTATGCAGTTGGCAGTAGAT
		ATCTACTGCCAACTGCATAGTGTCAACAGAAAGATGACA
c.571+1del	ivs3	ATAGCAGAAAAACACAAGGGAGAGGTAAGTTAGTAAATGATCTTCTTTTT
		AAAAAGAAGATCATTTACTAACTTACCTCTCCCTTGTGTTTTTCTGCTAT
c.572-3C>G	ivs3	TAAGAGTGTTTTGTTGTTTGAGAACACCGAAAAGCTTTG
		CAAAGCTTTTCGGTGTTCTCAAACAACAAAACACTCTTA
c.572-1G>C	ivs3	AGAGTGTTTTGTTGTTTCACAACACCGAAAAGCTTTGGA
		TCCAAAGCTTTTCGGTGTTGTGAAACAACAAAACACTCT
c.705+1G>A	ivs4	CCTTTCAGAACACTCAAAGATATGAGTCAGACTACTGAA
		TTCAGTAGTCTGACTCATATCTTTGAGTGTTCTGAAAGG
c.705+3A>G	ivs4	TTTCAGAACACTCAAAGGTGTGAGTCAGACTACTGAAAT
		ATTTCAGTAGTCTGACTCACACCTTTGAGTGTTCTGAAA
c.706-1G>T	ivs4	ATTATCTCTTCTGTATTTATGTTCGACTAGTGATAGTGG
		CCACTATCACTAGTCGAACATAAATACAGAAGAGATAAT
c.837+1G>T	ivs5	AAATAATCACAGATTAGCTTTAAGTATTAACTAGTGAAG
		CTTCACTAGTTAATACTTAAAGCTAATCTGTGATTATTT
c.837+4_837+7del	ivs5	TGCAAATAATCACAGATTAGCTGTATTAACTAGTGAAGAGAGTTTTATAA
		TTATAAAACTCTCTTCACTAGTTAATACAGCTAATCTGTGATTATTTGCA
c.838-2A>G	ivs5	TCTTACATTTTGTTTTTGTGGGTAATTTTAACCAATCAG
		CTGATTGGTTAAAATTACCCACAAAAACAAAATGTAAGA
c.904G>A	Ex6	CTTGCTTGTTCCTGCATTAAGTGGGTAATTAATCAGATA
		TATCTGATTAATTACCCACTTAATGCAGGAACAAGCAAG
c.904+1G>T	ivs6	TTGCTTGTTCCTGCATTAGTTGGGTAATTAATCAGATAA
		TTATCTGATTAATTACCCAACTAATGCAGGAACAAGCAA
c.905-3_906del	ivs6	ATACAGTTATTATGTTTTTTACTCTGAAAGTTGGGGACATGCTGCTACAA
		TTGTAGCAGCATGTCCCCAACTTTCAGAGTAAAAAACATAATAACTGTAT
c.905-2del	ivs6	TACAGTTATTATGTTTTTTACTCTCGGGGAAAGTTGGGGACATGCTGCTA
		TAGCAGCATGTCCCCAACTTTCCCCGAGAGTAAAAAACATAATAACTGTA
c.965+1G>A	ivs7	ATTGGGACCGAAAGCAAAGATCAGTACAGAAACAAGTTA
		TAACTTGTTTCTGTACTGATCTTTGCTTTCGGTCCCAAT
c.966-1G>C	ivs7	TATTCTTTTTCTTTAAGCACGTTGGCAACATTGTACAAG
		CTTGTACAATGTTGCCAACGTGCTTAAAGAAAAAGAATA
