# Supplementary material for: Minigene Splicing Assays Identify 20 Spliceogenic Variants of the Breast/Ovarian Cancer Susceptibility Gene RAD51C
Source: Cancers (Basel). 2022 Jun 15;14(12):2960. doi: 10.3390/cancers14122960 (PMC9221245; doi:10.3390/cancers14122960)
Supplement: Supplementary file 1 [file cancers-14-02960-s001.zip › Supplementary_Table_S3_Transcript_annotation.pdf]

Supplementary Table S3. Transcript annotation according to HGVS guidelines.

Transcripts	HGVS-RNA	HGVS-Protein
Δ(E2) [PTC-NMD]	r.146_404del	p.Glu49Valfs*6
Δ(E2p3)	r.146_148del	p.Glu49del
▼(E2q27)-a [PTC-NMD]	r.[404+2u>c,404_405ins404+1_404+27]	p.Cys135delinsTrpGlnAsnLysValPheSerPhe*
▼(E2q27)-b [PTC-NMD]	r.[404+3a>g,404_405ins404+1_404+27]	p.Cys135delinsTrp*
Δ(E2q175) [PTC-NMD]	r.230_404del	p.Gly77Valfs*6
Δ(E3p7) [PTC-NMD]	r.405_411del	p.Met136Trpfs*3
Δ(E3) [PTC-NMD]	r.405_571del	p.Cys135*
Δ(E3q1) [PTC-NMD]	r.571del	p.Glu191Asnfs*48
Δ(E4) [PTC-NMD]	r.572_705del	p.Glu191Glyfs*16
Δ(E5p10) [PTC-NMD]	r.706_715del	p.Val236*
Δ(E5)	r.706_837del	p.Arg237_Val280del
Δ(E6) [PTC-NMD]	r.838_904del	p.Val280Glyfs*12
Full-length-c.904G>A	r.904g>a	p.Gly302Arg
▼(E6q4)-a [PTC]	r.[904g>a,904_905ins904+1_904+4]	p.Gly302Serfs*47
▼(E6q4)-b [PTC]	r.[904+1g>u,904_905ins904+1_904+4]	p.Gly302Valfs*47
Δ(E7) [PTC]	r.905_965del/	p.Glu303Trpfs*41
Δ(E8) [PTC]	r.966_1026del	p.Arg322Serfs*22
▼ (E8p3)	r. [966-1g>c,965_966ins966-3_966-1]	p.Arg322delinsSerThr
